# Analysis of Atrial Fibrillation Treatment Regimes in a Multicenter Cohort of Transcatheter Edge-to-Edge Mitral Valve Repair Patients

**DOI:** 10.1155/2020/6542028

**Published:** 2020-08-28

**Authors:** Christian Waechter, Felix Ausbuettel, Georgios Chatzis, Dieter Fischer, Holger Nef, Sebastian Barth, Philipp Halbfaß, Thomas Deneke, Sebastian Kerber, Dimitar Divchev, Bernhard Schieffer, Ulrich Luesebrink

**Affiliations:** ^1^Department of Cardiology, University Hospital, Marburg, Germany; ^2^Department of Cardiology, Cardiovascular Center, Rotenburg, Fulda, Germany; ^3^Department of Cardiology, University Hospital, Giessen, Germany; ^4^Department of Cardiology, Cardiovascular Center, Bad Neustadt, Saale, Germany

## Abstract

**Background:**

Atrial fibrillation (AF) is a highly prevalent comorbidity in patients with severe mitral valve regurgitation (MR). Recent studies show a deleterious outcome of patients with concomitant AF after transcatheter mitral valve repair (TMVR). This underlines the essential need for additional strategies that ameliorate the prognosis of these patients. Fundamental data on AF characteristics and treatment regimes in this special cohort of patients are lacking.

**Methods:**

We retrospectively analyzed the data of 542 consecutive patients with severe MR undergoing TMVR in three tertiary heart centers with special focus on AF type and underlying treatment strategies.

**Results:**

The prevalence of concomitant AF was 73.3%, and AF did not affect the procedural success or the incidence of major adverse cardiac and cerebrovascular events. The patients with AF were more frequently >75 years, had more tricuspid regurgitation, and less coronary artery disease than non-AF patients. The distribution of AF types was 32% paroxysmal AF, 27% persistent AF, and 41% permanent AF. Except for a higher degree in severe tricuspid regurgitation and a higher likelihood of male sex, no substantial differences were observed while comparing permanent and nonpermanent AF patients. The predominant treatment regime was rate control (57%), with only beta blockers (BB) in the majority of persistent and permanent AF patients, while additional digitalis or a pacemaker was used infrequently. Rhythm control was mainly achieved with BB alone in paroxysmal AF patients and with additional antiarrhythmic drugs in the majority of persistent AF patients. Interventional rhythm control therapy was performed in 2.5% and 30.9% of paroxysmal and persistent AF patients, respectively. The guideline-adherent use of oral anticoagulants was comparable and high in both groups (91.9% in nonpermanent vs. 90.1% in permanent AF).

**Conclusion:**

This is the first study to provide necessary information for the understanding of the current clinical practice in dealing with TMVR patients. Since evidence suggests that AF is not a benign concomitant disease, further investigations are needed to assess the prognostic impact of these different AF treatment strategies.

## 1. Introduction

The percutaneous edge-to-edge repair with the MitraClip® device (Abbott Vascular, Santa Clara, CA, USA) has been established as a safe and efficacious therapy for severe mitral regurgitation (MR) and is widely offered to a growing number of patients who are ineligible candidates for cardiac surgery. Reflecting the complex pathophysiological interplay, atrial fibrillation (AF) is a common comorbidity in this cohort of patients, real-world data reporting AF prevalence of up to 70.2% with a deleterious impact on the outcome after transcatheter mitral valve repair (TMVR) [1]. Thus, recently published studies show a higher risk for bleeding and stroke, higher rates of heart failure hospitalizations, and finally a higher mortality of patients with concomitant AF undergoing TMVR [2–4]. This implies the essential need for additional strategies ameliorating the prognosis of these patients. Nevertheless, fundamental data on current AF treatment regimes in this special cohort of patients are lacking. The present study sheds light on cardiac rhythm management strategies in an all-comer multicenter population that underwent percutaneous edge-to-edge mitral valve repair.

## 2. Methods

Data from all consecutive patients scheduled for percutaneous therapy of MR using the MitraClip® device in three tertiary heart centers in Germany between October 2011 and October 2019 were retrospectively collected and analyzed. All patients were examined by the particular local interdisciplinary heart team consisting of interventional cardiologists and cardiac surgeons and were adjudged to interventional therapy due to high surgical risk for conventional surgery. The procedures were performed by certified and experienced operators according to the manufacturer's instructions either in general anesthesia or analgosedation only as described previously [5]. In the aforementioned period, all treated patients were identified and the corresponding data of the baseline hospitalization were collected using the clinical information system of each participating center. Besides a wide range of clinical characteristics, the study population in particular was evaluated for the history of atrial fibrillation (paroxysmal, persistent, or permanent) and the underlying therapeutic concept of either rate or rhythm control with the related medication or intervention, respectively. The types of AF have been defined according to the currently valid guidelines of the European Society of Cardiology [6]. In brief, paroxysmal AF was defined if an episode lasted a maximum of seven days, regardless of the modality of its termination. Accordingly, persistent AF was defined, if an episode lasted longer than seven days with or without cardioversion after this period of time and a rhythm control strategy was adopted. Due to partially incomplete medical recordings, we cannot differentiate between persistent and longstanding persistent AF. Thus, all patients with AF episodes that lasted longer than seven days or were terminated after seven days were defined as “persistent AF.” If rhythm control interventions were not pursued anymore, AF was defined as permanent and therefore the corresponding treatment concept defined as rate control. Furthermore, we defined all the examined patients with paroxysmal AF to be treated with the objective of rhythm control. The patients with persistent AF status were defined to be on rate control if the medication consists of beta blockers only or a combination of beta blockers and digitalis or if a pacemaker has been implanted with or without an additional AV node ablation. Rhythm control was assumed to be intended, if patients with persistent AF were treated with antiarrhythmic drugs other than beta blockers or if pulmonary vein isolation (PVI) was performed prior TMVR. The local ethics committee approved the study.

All statistical analyses were done by using *R* (*R* Foundation for Statistical Computing, Vienna, Austria), SPSS 24.0 (IBM Corp., Armonk, NY, USA), and GraphPad Prism 6.0 (GraphPad Software, La Jolla, CA, USA). Categorical variables are presented as frequencies and percentages (%), and continuous variables are presented as mean and standard deviation for standard distributed variables and median and interquartile ranges (IQR: 25^th^–75^th^ percentile) for nonstandard distributed variables. A two-sided *p* value of <0.05 was considered statistically significant. Differences between two groups were compared using the chi-squared test and Fisher's exact test for categorical variables and Student's *t*-test for standard distributed variables and the Wilcoxon test for nonstandard distributed variables. In case of three or more groups, differences were compared using analysis of variance (ANOVA) for standard distributed variables and the Kruskal–Wallis test for nonstandard distributed variables.

## 3. Results

We identified 542 consecutive patients with severe MR who were scheduled for TMVR using MitraClip®. Thirty-six patients (6.6%) did not accomplish the procedure successfully and were excluded from further analysis. Among these nonsuccessfully treated patients, 63.9% had a history of AF. Here, the concomitance of AF correlated not with the success of TMVR procedure (OR, 0.62, 95% confidence interval, 0.29–1.39; *p*=0.27).

In the analyzed cohort, 373 (73.3%) patients revealed a history of AF. In comparison with patients without a known history of AF, the AF patients were more frequently >75 years old (74.3% vs. 67%, *p*=0.02) and had a lower prevalence of concomitant coronary heart disease (63.3% vs. 77.4%, *p*=0.003). Thus, patients with a history of AF obtained fewer percutaneous coronary interventions (52.5% vs. 62.4%, *p*=0.04) and aortocoronary bypass surgery (23.6% vs. 35.3%, *p*=0.008). Furthermore, patients with a history of AF suffered more frequently from severe tricuspid regurgitation (21.4% vs. 11.3%, *p*=0.0006). There were no statistically significant differences between patients without AF and with permanent and nonpermanent AF regarding other clinical and procedural characteristics. No substantial differences were observed regarding the congestive heart failure medication between the groups. However, the significantly more frequent use of digitalis in the permanent AF group compared with the nonpermanent AF patients (17.8% vs. 5.4%, *p* ≤ 0.0006) and the slight use of ivabradine in the non-AF compared with nonpermanent and permanent AF group (3.8% vs. 0.9% vs. 0%, *p*=0.023) consisted an exception.

Regarding the incidence of major adverse cardiac and cerebrovascular events (MACCE) and in-hospital death from any cause, no statistically significant differences were observed between patients with nonpermanent AF, with permanent AF, and without a history of AF (5.9% vs. 4.6% vs. 8.3%, *p*=0.43; 3.6% vs. 4.0% vs. 4.5%, *p*=0.92). The clinical and procedural data and the distribution of the heart failure medication are summarized in Tables 1 and 2, respectively.

Among the 373 patients revealing history of AF, the majority of 152 (41%) patients were defined as permanent AF. One hundred nineteen patients had paroxysmal AF (32%), and 102 patients (27%) were classified as persistent AF. Patients with permanent AF were more likely to be male (71.0% vs. 58.4%, *p*=0.01) and suffered more often from concomitant severe tricuspid regurgitation (29.6% vs. 15.8%, *p*=0.001) compared with patients with nonpermanent AF. As far as the comparison between paroxysmal and persistent AF patients is concerned, there were no statistically significant differences in clinical and procedural characteristics observed and therefore not shown.

Corresponding to the current guidelines [6, 7], all analyzed patients with a history of AF had the indication for an oral anticoagulation as the calculated CHA_2_DS_2_-VASc score was 5.0 ± 1.35 in patients with nonpermanent AF and 5.1 ± 1.34 (*p*=0.44) in patients with permanent AF. The use of anticoagulating medication in total was high in both the groups (91.9% in nonpermanent AF vs. 90.1% in permanent AF, *p*=0.56). A significantly higher rate of patients with permanent AF received vitamin K antagonists (61.8% vs. 42.9% vs. 49.0%, *p*=0.01) and less frequent direct oral anticoagulants (DOAC) (28.3% vs. 47.9% vs. 44.2%, *p*=0.01) than paroxysmal and persistent AF patients, accordingly. Except for the above-mentioned higher prevalence of male sex in the permanent AF group, there was no statistically significant difference in the composition of other parameters of the CHA_2_DS_2_-VASc score.

Following the above-mentioned definitions, the majority of patients analyzed was on rate control (57%), and if considering the persistent AF cohort, rate control was in 60/102 patients (59%) the underlying treatment concept. The vast majority of patients on rate control was treated with beta blockers (90.0% vs. 80.9%, *p*=0.25) in both the groups—persistent and permanent AF. Pacemaker implantation with or without additional AV node ablation (21.7% vs. 10.0%, *p* ≤ 0.001) and the combination of beta blockers and digitalis (11.8% vs. 3.3%, *p* ≤ 0.001) were used significantly more often in the permanent than persistent AF group.

**Figure 1:**
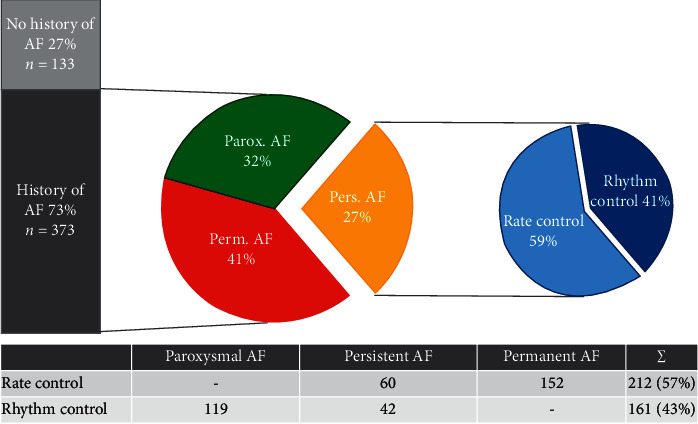
AF type and corresponding treatment regimes. Overview of patients with and without a history of AF, AF types, and corresponding treatment regimes. AF, atrial fibrillation; Parox., paroxysmal; Pers., persistent; Perm., permanent.

Rhythm control was intended in 161/373 patients (43%). In paroxysmal AF, rhythm control therapy predominantly consisted of beta blockers (66%) followed by the combination of beta blockers and amiodarone, the exclusively used class III antiarrhythmic drug (AAD) in the studied cohort. The use of other classes of AADs (0.8% class I, 0.0% class IV) or pulmonary vein isolation (PVI, 2.5%) was insignificant. In the persistent AF group, rhythm control was aspired significantly more often to the use of beta blockers plus amiodarone (54.8% vs. 27.7%, *p*=0.0024), PVI (30.9% vs. 2.5%, *p* ≤ 0.0001), or amiodarone alone (16.7% vs. 2.5%, *p*=0.0034) compared with the paroxysmal AF group.  Figure 1 summarizes the distribution of the AF treatment strategies in the examined cohort, and Table 3 shows the anticoagulation as well as the corresponding medication and interventions used, accordingly.

## 4. Discussion

Reflecting the complex pathophysiological interplay, AF is one of the most common comorbidities in severe MR and is associated with a deleterious impact on the outcome after transcatheter mitral valve reconstruction (TMVR). This implies the essential need for additional strategies that ameliorate the prognosis of patients with AF undergoing TMVR. Therefore, profound information about the current clinical practice in handling AF in this special cohort of patients is required. Here, we provide an in-depth analysis of AF types, characteristics, and treatment strategies in one of the largest German multicenter cohorts of patients with severe MR who underwent edge-to-edge TMVR.

Compared with recent trials and registries, which report the prevalence of concomitant AF to be 27% to 70.2%, our study reveals the highest prevalence of AF (73.3%) in a TMVR cohort to date [1–4, 8–10]. Regarding MR etiology, procedural characteristics, technical success, reduction of MR, and periprocedural mortality, we achieve to a high extent similar results, reflecting the comparability of our studied cohort to that of the last-mentioned published data. Thus, we confirm that concomitant AF does not affect the safety and success of TMVR nor has it a negative impact on the outcome during procedural hospitalization.

In line with the published data, the patients with AF were more often >75 years old and predominantly male. Similar to the observation of Arora et al. and Velu et al., the patients with AF had ischemic heart disease less frequently and thus received fewer previous percutaneous coronary interventions and cardiac bypass surgery [2, 4].

Furthermore, we also observed a higher proportion of patients with concomitant severe tricuspid regurgitation in the group of AF patients, confirming another observation of Velu et al. and Keßler et al. [2, 3]. In our cohort, in particular, in patients showing permanent AF compared with other AF types, a significantly higher rate of concomitant severe TR was exhibited. Even if data on atrial diameters or tricuspid annular diameters are not available for analysis, it is known that in AF patients, especially with longstanding type, are prone to biatrial enlargement, resulting in functional regurgitation of the atrioventricular valves [11, 12]. This in turn seems to predict the likelihood of surgical valve repair, in particular if both severe MR and concomitant severe TR are present [13].

Regarding the prevention of thromboembolism, as it has pivotal impact on morbidity and mortality, we observed the use of anticoagulants in 91.9% in nonpermanent AF and in 90.1% in permanent AF (Table 3). This represents a very high level of guideline-adherent use of anticoagulants compared with the levels ranging from 62.6% to 72.6% reported from “real-world” data and ranging up to 88.8% in trials like COAPT [3, 4, 14]. At this point, it has to be noted that only a very low proportion of studies report the rates of anticoagulants used. The predominantly used agents in our collective were vitamin K antagonists with a significantly higher use in the group of permanent AF patients compared with the nonpermanent AF groups. This could partly be explained by a long-term use of vitamin K antagonists, which was started before the advent of the DOACs and furthermore by the lesser clinical experience and the less clear guideline recommendations regarding the DOACs at the beginning of the analyzed period. In addition, the rarely occurring confusion among prescribers about the term “valvular AF,” which is a contraindication for DOACs only applying to relevant mitral stenosis and not to regurgitation, could have made a further contribution.

We observed a very high degree of guideline adherence in our studied population regarding the concomitant heart failure medication. Thus, no substantial differences can be seen, when compared exemplarily with the COAPT trial in which persistence of heart failure symptoms despite maximal guideline-directed medical therapy was the main inclusion criterion for TMVR [14].

Taking all AF types together as well as considering only the persistent AF group, the vast majority of the patients studied were on rate control therapy. This was largely achieved through the usage of beta blockers and through the combination of beta blockers and digitalis, which were used significantly more frequently in the group of permanent AF. Rhythm control, on the other hand, consisted mainly of the combination of amiodarone and beta blockers in the group of persistent AF. This could be explained by the significantly higher safety profile of amiodarone compared with Class I AADs particularly in light of concomitant structural heart disease [15]. An interventional strategy of rhythm control generally played only a subordinate role, especially in paroxysmal AF patients. Here, the recent demonstration of feasibility, efficacy, and safety of pulmonary vein isolation after prior TMVR by Rottner et al. could lead to increased usage of this strategy even in this cohort of patients [16]. Regarding the prognostic effect of this intervention, due to the small number of 14 patients, no reliable statements can be made, which ultimately highlights the need for further studies.

In general, there is paucity of data and controversial debates constructed on atrial fibrillation therapy in elderly patients, exactly as in our analyzed cohort showing a mean age of 78.4 years. The current European and US guidelines, which have been updated recently, still suggest rhythm control strategies predominantly for those patients who remain symptomatic despite adequate rate control [6, 7]. Even if no age is specified, these recommendations are aiming at younger patients with fewer comorbidities. However, who can surely exclude that heart failure symptoms in patients with severe MR that are present preprocedural or even remain after TMVR do not result from coexisting atrial fibrillation? Beyond that, a data analysis from the European Heart Survey by Fumagalli et al. revealed that elderly patients less often receive rhythm control therapy, even when presenting with symptoms [17]. It is therefore not surprising that according to the “real-world” EORP-AF registry data, rate control is the predominant therapy concept for advanced age patients [18]. Although there is evidence for a lower rate of cerebral ischemia as well as higher health-related quality of life among rhythm-controlled patients [19, 20], contrary evidence is provided by a subgroup analysis of the AFFIRM study, which due to the lack of robust newer data continues to be relevant. Here, Shariff et al. showed a significantly lower mortality in patients between 70 and 80 years of age with rate control vs. rhythm control [21]. However, due to some methodological weaknesses of the AFFIRM study, like ceasing anticoagulation in rhythm-controlled patients and the lack of consideration of interventional rhythm control therapy, the results of the study should only be transferred carefully to today's patient populations.

Finally, the most important question remains: Does rhythm control therapy ameliorate the prognosis of this cohort of patients? Our study, which only examines retrospectively the situation at baseline, is obviously unable to answer this question. If the evidence from patients who underwent surgical mitral valve reconstruction with concomitant ablation of atrial fibrillation or the evidence from the CASTLE-AF trial is paralleled to the TMVR collective, a benefit could be hypothesized [22–24].

There are several limitations of our study that have to be emphasized. First, the analyzed data were collected retrospectively. Hereby, we cannot provide detailed echocardiographic or hemodynamic parameters due to incomplete data collection. Furthermore, we cannot assess the effectiveness of the AF therapies because relevant data were not gathered.

In summary, it can be said that AF is a highly prevalent comorbidity in the “real-world” TMVR patients. Except for a higher prevalence of concomitant severe tricuspid regurgitation, no relevant and fundamental differences compared with patients without atrial fibrillation can be found. The vast majority of these patients are treated with respect to rate control using beta blockers. Rhythm control is achieved mainly with amiodarone and a combination of amiodarone and beta blockers. Invasive rhythm control strategies are infrequently used and of subordinate relevance. Here, we provide clinical insights that are necessary for the understanding of the actual clinical practice in dealing with TMVR patients. Since current evidence suggests that AF is not a benign concomitant disease, further investigations are required to assess the prognostic impact of these different AF treatment strategies.

## Figures and Tables

**Table 1:** Clinical characteristics and heart failure medication.

	Total, *n* = 506	Non-AF, *n* = 133	Permanent AF, *n* = 152	Nonpermanent AF, *n* = 221	*p* value
Age (years)	78.1 ± 7.8	77.4 ± 9.1	79.1 ± 6.9	77.9 ± 7.6	0.2926
Male sex	62.9%	60.9%	71.1%	58.4%	**0.012**
EuroSCORE II (IQR)	20.0% (23.0)	19.1% (21.9)	20.1% (22.8)	19.6% (23.3)	0.74
STS risk score (IQR)	7.4% (8.8)	7.3% (8.9)	8.5% (10.1)	6.7% (8.5)	0.5924
NYHA class I NYHA class II NYHA class III NYHA class IV	0.2% 4.7% 71.0% 24.1%	0.0% 5.3% 73.7% 21.0%	0.0% 3.9% 70.4% 25.7%	0.5% 5.0% 69.6% 24.9%	0.71
Chronic obstructive pulmonary disease	20.6%	22.6%	23.0%	17.7%	0.5
Coronary artery disease	67.0%	77.4%	64.5%	62.4%	**0.0028**
Prior cardiac bypass surgery	26.7%	35.3%	25.0%	22.6%	**0.0085**
Prior percutaneous coronary intervention	55.1%	62.4%	48.7%	55.2%	**0.049**
Diabetes mellitus	33.4%	35.3%	36.2%	30.3%	0.69
Art. hypertension	80.0%	79.7%	81.6%	79.2%	0.91
Prior stroke	10.9%	11.3%	11.2%	10.4%	0.86
Preexisting ICD	26.9%	26.3%	25.7%	28.1%	0.94
Preexisting CRT	11.7%	11.3%	9.2%	13.6%	0.43
Glomerular filtration rate (mL/min)	47.4 ± 20.2	50.0 ± 22.0	47.9 ± 20.0	45.5 ± 19.2	0.1
NT-pro BNP (ng/L)	2 945.0 ± 4 902.0	2 960.0 ± 5 871.0	2 921.0 ± 4 687.0	2 951.0 ± 4 304.0	0.41
LV function > 45% LV function 30–44% LV function < 30%	38.7% 34.6% 26.7%	30.1% 38.3% 31.6%	44.1% 33.6% 22.3%	40.3% 33.0% 26.7%	0.053
TR grade III	18.8%	11.3%	29.6%	15.8%	**0.0006**
Degenerative MR etiology Functional MR etiology Combined MR etiology	27.5% 64.6% 7.9%	27.1% 65.4% 7.5%	26.3% 65.8% 7.9%	28.5% 63.4% 8.1%	0.96

Heart failure medication					
ACE/AT1 inhibitors	74.1%	72.9%	72.4%	76.2%	0.6
ARN inhibitor	7.9%	7.5%	8.6%	7.7%	0.93
Beta blockers	87.9%	87.2%	87.5%	88.7%	0.72
Loop diuretics	89.5%	87.2%	90.8%	90.1%	0.33
Thiazide diuretics	21.3%	21.8%	25.7%	18.1%	0.21
Aldosterone antagonists	48.0%	47.4%	47.4%	48.9%	0.91
Ivabradine	1.4%	3.8%	0.0%	0.9%	**0.023**
Digitalis	7.7%	0.0%	17.8%	5.4%	**<0.0001**

Data presented as mean ± SD or median with interquartile range (IQR). AF, atrial fibrillation; ICD, implantable cardioverter defibrillator; CRT, cardiac resynchronization therapy; TR, tricuspid regurgitation; MR, mitral regurgitation; ACE, angiotensin-converting enzyme; AT1, angiotensin II type 1 receptor; ARN, angiotensin receptor neprilysin. *p* values describe differences between patients without AF and with permanent and nonpermanent AF. The bold values indicate the significance of *p* values.

**Table 2:** Procedural characteristics.

	Total,	Non-AF,	Permanent AF,	Non-permanent AF,	*p*- value
	*n* = 506	*n* = 133	*n* = 152	*n* = 221	
Mean procedure duration (min)^*∗*^	108.2 ± 63.1	104.0 ± 53.0	116.7 ± 74.5	104.8 ± 59.6	0.13

Postinterventional no MR	24.7%	23.3%	22.4%	27.1%	0.58
Postinterventional MR grade I	62.9%	62.4%	62.5%	62.3%	
Postinterventional MR grade II	12.0%	13.5%	14.5%	9.5%	
Postinterventional MR grade III	0.4%	0.8%	0.7%	0.0%	

1 clip implanted	37.9%	36.8%	41.5%	36.2%	0.41
2 clips implanted	52.8%	51.9%	48.0%	56.6%	
3 clips implanted	9.1%	11.3%	10.5%	6.8%	
4 clips implanted	0.2%	0.0%	0.0%	0.5%	

Length of hospital stay (days)	7.0	7.0	7.0	7.0	0.27
IQR Length of hospital stay	5.0	5.0	6.3	5.0	

MACCE	4.4%	5.3%	2.0%	5.4%	0.27
(i) cerebral/systemic thromboembolic event	0.6%	0.8%	0.0%	0.9%	0.62
(ii) bleeding requiring intervention/transfusion	2.2%	3.0%	2.0%	1.8%	0.74
(iii) In-hospital death from cardiovascular cause	1.6%	1.5%	0.0%	2.7%	0.11

In-hospital death from any cause	4.2%	4.5%	3.3%	4.5%	0.82

^*∗*^Data presented as mean ± SD. MR, mitral regurgitation; IQR, interquartile range; MACCE, major adverse cardiac and cerebrovascular events (including cerebral or systemic thromboembolic event, bleeding that requires intervention or transfusion, and in-hospital death from cardiovascular cause). *p* values describe differences between patients without AF and with permanent and nonpermanent AF.

**Table 3:** Anticoagulation, CHA_2_DS_2_-VASc score, and AF treatment regimens with corresponding medication and interventions.

	Paroxysmal AF, *n* = 119	Persistent AF, *n* = 102	Permanent AF, *n* = 152	*p* value
Anticoagulant				
Vitamin K antagonist	42.9%	49.0%	61.8%	**0.01**
Direct oral anticoagulants	47.9%	44.2%	28.3%	**0.01**
CHA_2_DS_2_-VASc score^*∗*^	5.1 ± 1.4	4.9 ± 1.3	5.1 ± 1.3	0.5

Rate control		*n* = 60		
Class II AAD	—	90.0%	80.9%	0.25
Class II AAD + digitalis	—	3.3%	11.8%	**<0.001**
Pacemaker ± AVN ablation	—	10.0%	21.7%	**<0.0001**

Rhythm control		*n* = 42		
Class I AAD	0.8%	0.0%	—	1
Class II AAD	58.0%	—	—	—
Class III AAD	2.5%	16.7%	—	**0.0034**
Class IV AAD	0.0%	0.0%	—	1
Class II + class III AAD	27.7%	54.8%	—	**0.0024**
Pulmonary vein isolation	2.5%	30.9%	—	**<0.0001**

^*∗*^Data presented as mean ± SD. AF, atrial fibrillation; AAD, antiarrhythmic drug; AVN, AV node. *p* values describe differences between patients with paroxysmal, persistent, and permanent AF. The bold values indicate the significance of p values.

## Data Availability

The data used to support the findings of this study are available from the corresponding author upon request.
